# Supplementary material for: Water-powered self-propelled magnetic nanobot for rapid and highly efficient capture of circulating tumor cells
Source: Commun Chem. 2021 Nov 18;4:159. doi: 10.1038/s42004-021-00598-9 (PMC9814645; doi:10.1038/s42004-021-00598-9)
Supplement: Supplementary file 1 — Supplementary Information [file 42004_2021_598_MOESM1_ESM.pdf]

Water-Powered Self-Propelled Magnetic Nanobot for Rapid and Highly Efficient Capture of Circulating Tumor Cells

Supplementary Information

Ravindra D. Wavhale,[1] Kshama D. Dhobale,[1] Chinmay S. Rahane,[1] Govind P. Chate,[1]

Bhausaheb V. Tawade,[1] Yuvraj N. Patil,[1] Sandesh S. Gawade[2] and Shashwat S. Banerjee*[1]

[1]Central Research Laboratory, Maharashtra Institute of Medical Education and Research, Talegaon Dabhade, Pune 410507, India

[2]Department of Surgery, Maharashtra Institute of Medical Education and Research, Talegaon Dabhade, Pune 410507, India

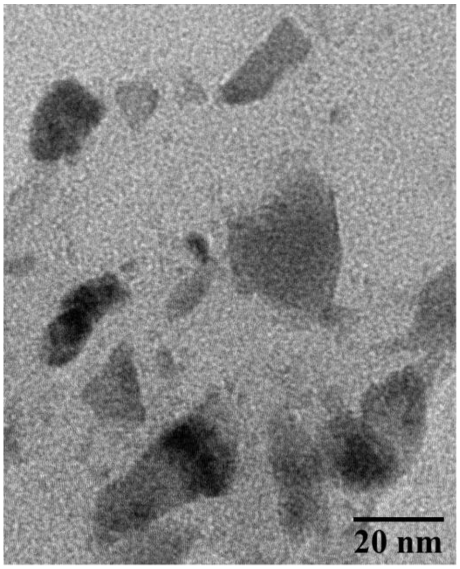

Supplementary Figure S1. TEM image of MFN.

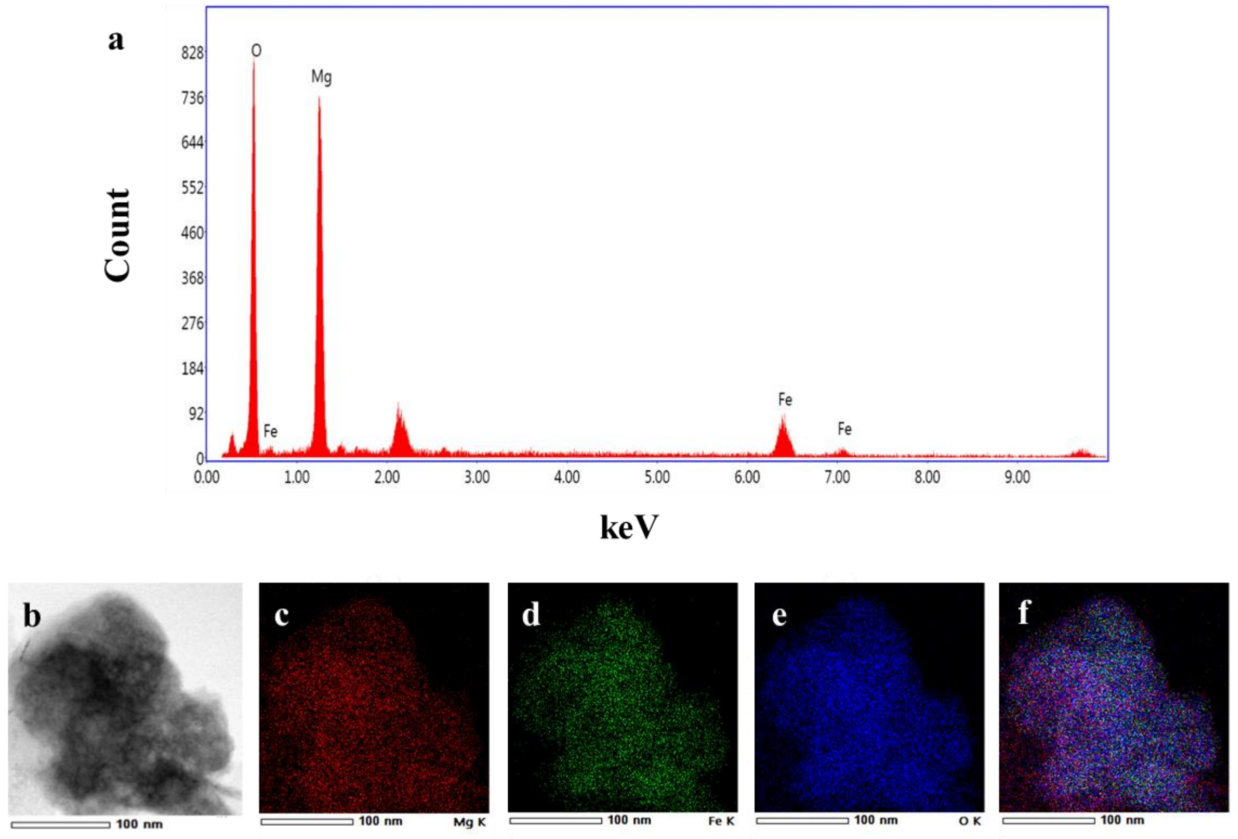

Supplementary Figure S2. a, STEM-energy dispersive X-ray spectrum (EDX) of MFN showing magnesium (Mg), iron (Fe), and oxygen (O). **b-f**, Scanning transmission electron microscopy (STEM) image of MFN with corresponding elemental maps.

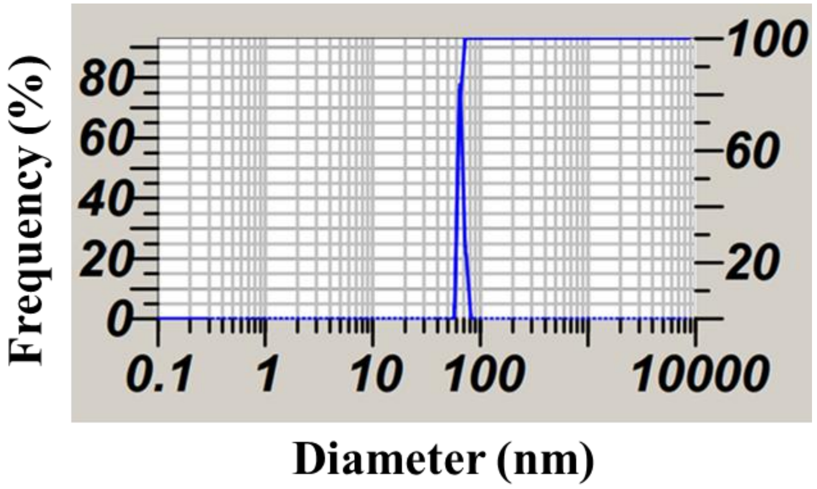

Supplementary Figure S3. Dynamic light scattering (DLS) size distribution curve of MFN.

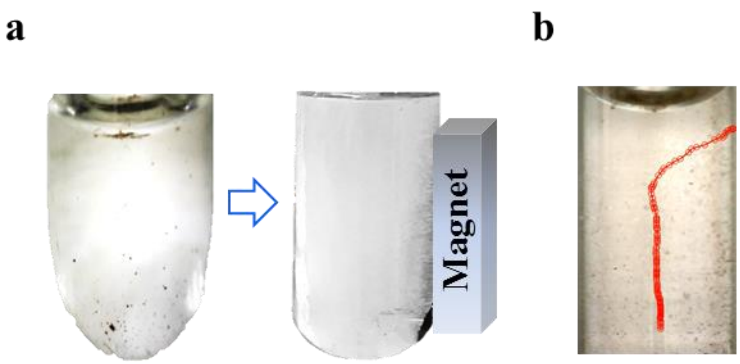

Supplementary Figure S4. a, MFN are drawn from the solution to the sidewall of the vial when exposed to a magnetic field due to the presence of Fe_3O_4 hemispherical shell. **b,** Tracking trajectories of autonomously propelling MFN when exposed to a magnetic field.

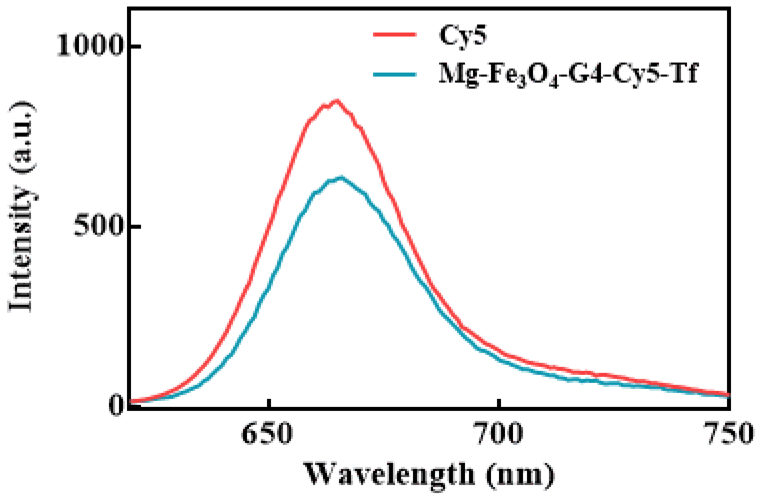

Supplementary Figure S5. Normalized fluorescence emission spectra of Cy5 and MFN in PBS.

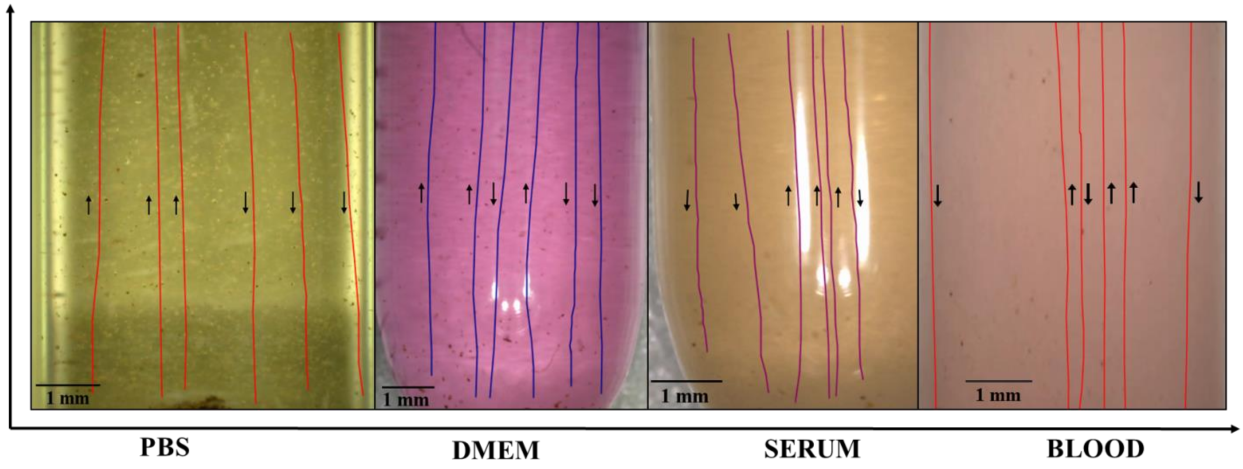

Supplementary Figure S6. Analysis of the motion behavior of MFN. Representative tracking trajectories of MFNs in different biologically relevant media.

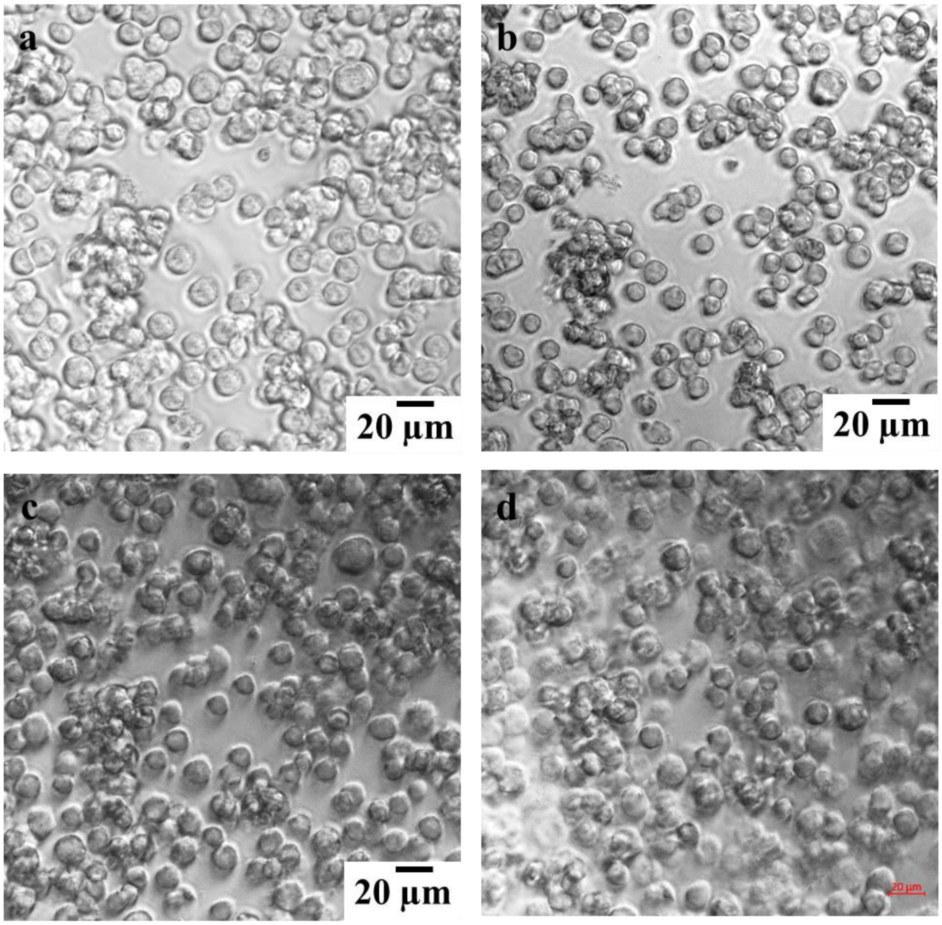

Supplementary Figure S7. Effect of $NaHCO_3$ on HCT116 cells at **a,** 0 min, **b,** 10 min, **c,** 30 min and **d,** 60 min. $NaHCO_3$ does not appear to purturb cell significantly as seen in the images.

Supplementary Table S1. Total propulsion time of MFN in various media.

	MNF propulsion time (min)	
**$NaHCO_3$ Conc (M).**	**0**	**0.5**
DMEM	1.1	5.2
PBS	0	5.8
Serum	7	>28

Supplementary Table S2. Number of HCT116 and MCF7 cells captured with MFNs in various biological media.

Cells Added	NanoSystem Used								Media
	MFN (Tf)		MFN (Ab)		MFN (Tf) + $NaHCO_3$		MFN (Ab) + $NaHCO_3$		
	% Cells Captured								
	HCT116	MCF7	HCT116	MCF7	HCT116	MCF7	HCT116	MCF7	
10	25	29.17	40	45.83	100	100	100	100	DMEM
25	29.17	50.00	31.25	55.36	100	100	100	100	
50	48.04	36.44	46.08	39.83	99.04	100	99.04	100	
75	46.58	42.11	43.84	48.68	97.95	100	97.95	100	
100	37.86	47.14	35.92	50.00	93.81	100	93.30	100	
10	36.11	39.29	37.5	32.14	100	100	100	100	PBS
25	38.37	57.14	36.67	48.22	100	100	100	100	
50	55.67	55.66	52.54	47.17	95.69	100	100	100	
75	31.08	51.32	35.14	55.92	96.53	100	95.14	100	
100	25.21	51.38	29.21	40.83	92.07	100	91.01	100	
10	50	68.75	44.44	50	100	100	100	100	Blood
25	66.67	56.90	64.81	41.38	100	100	100	100	
50	43.86	50.00	39.47	38.46	98.18	100	99.09	100	
75	56.76	51.33	41.22	44.00	99.31	100	98.61	100	
100	50.49	54.95	56.80	48.52	98.44	100	98.96	100	
10	40	59.09	35	50	100	100	100	100	Lysed Blood
25	58.93	52.00	41.07	38	100	100	100	100	
50	54.81	54.81	66.35	35.58	98.98	100	98.98	100	
75	41.22	52.00	45.95	45.33	98.70	100	98.70	100	
100	45.75	63.27	44.81	50	97.89	100	98.86	100	

Supplementary Table S3. Number of cancer cells captured by recent typical nanomaterial-based CTC isolation approaches.

Sr. No	Nanoparticle	Cell line used for testing	Cell count utilized for capture efficiency	Incubation Time	Capture efficiency	Reference
1	Anti-EpCAM-modified F-MNPs and dual (EpCAM-modified and anti-N-cadherin-modified) antibody-modified F-MNPs	MCF-7	10-200 cells/mL	>20 min	97% with Anti-EpCAM-modified F-MNPs and 98.8% with dual antibody-modified F-MNPs	[1]
2	Anti-EpCAM antibody modified RBC-IMNs (Immunomagnetic micro/nanoparticles)	MCF-7 and PC-3	10-200 cells/mL	0-24 h	~90% in PBS, ~60% in blood	[2]
3	MNPs@hydrogel-anti-EpCAM nanoparticles	MCF-7	5-100 cells/mL	25 min	97% in PBS and 96% in blood	[3]
4	Gelatin nanoparticle-coated silica microbead functionalized with anti-EpCAM and anti-CD146 Abs	MCF-7, MDA-MB-231, HCT116 and HT-29	20-250 cells/mL	20 min	>80%	[4]
5	Hollow glass microspheres modified anti-EpCAM antibody	MCF7	30-1000 cells/ml in 5x diluted blood	20 min	93.6%	[5]

Supplementary Table S4. CTCs captured by MFN in clinical samples of epithelial cancer patients.

Cancer patient data for clinical trials		
**Sr. No.**	**Cancer type**	**Number of CTCs mL^{-1} observed**
**1**	Colorectal	0
**2**	Breast	3
**3**	Lung	0
**4**	Breast	5
**5**	Ovarian	1

Supplementary References

1 Wang, Z. *et al.* High-Efficiency Isolation and Rapid Identification of Heterogeneous Circulating Tumor Cells (CTCs) Using Dual-Antibody-Modified Fluorescent-Magnetic Nanoparticles. *ACS Appl Mater Interfaces* **11**, 39586-39593, (2019).

2 Meng, Q.-F. *et al.* Biomimetic immunomagnetic nanoparticles with minimal nonspecific biomolecule adsorption for enhanced isolation of circulating tumor cells. *ACS applied materials & interfaces* **11**, 28732-28739 (2019).

3 Wang, Z. *et al.* Antifouling hydrogel-coated magnetic nanoparticles for selective isolation and recovery of circulating tumor cells. *Journal of Materials Chemistry B* **9**, 677-682 (2021).

4 Huang, Q. *et al.* Gelatin nanoparticle-coated silicon beads for density-selective capture and release of heterogeneous circulating tumor cells with high purity. *Theranostics* **8**, 1624 (2018).

5 Dong, Z. *et al.* Enhanced capture and release of circulating tumor cells using hollow glass microspheres with a nanostructured surface. *Nanoscale* **10**, 16795-16804 (2018).
