# Supplementary material for: Water-powered self-propelled magnetic nanobot for rapid and highly efficient capture of circulating tumor cells
Source: Commun Chem. 2021 Nov 18;4:159. doi: 10.1038/s42004-021-00598-9 (PMC9814645; doi:10.1038/s42004-021-00598-9)
Supplement: Supplementary file 2 — Description of Additional Supplementary Files [file 42004_2021_598_MOESM2_ESM.pdf]

# Description of Additional Supplementary Files

**File Name:** Supplementary Movie 1

**Description**: Propulsion of nanorobots by generation of bubbles
